# Metal Organic Framework-Incorporated Three-Dimensional (3D) Bio-Printable Hydrogels to Facilitate Bone Repair: Preparation and In Vitro Bioactivity Analysis

**DOI:** 10.3390/gels9120923

**Published:** 2023-11-23

**Authors:** Cho-E Choi, Aishik Chakraborty, Hailey Adzija, Yasmeen Shamiya, Khaled Hijazi, Ali Coyle, Amin Rizkalla, David W. Holdsworth, Arghya Paul

**Affiliations:** 1 Department of Chemical and Biochemical Engineering, The University of Western Ontario, London, ON N6A 5B9, Canada; 2 Collaborative Specialization in Musculoskeletal Health Research and Bone and Joint Institute, The University of Western Ontario, London, ON N6A 5B9, Canada; 3 Department of Chemistry, The University of Western Ontario, London, ON N6A 5B9, Canada; 4 School of Biomedical Engineering, The University of Western Ontario, London, ON N6A 5B9, Canada; 5 Department of Medical Biophysics, The University of Western Ontario, London, ON N6A 5B9, Canada; 6 Dentistry, The University of Western Ontario, London, ON N5A 5B9, Canada

**Keywords:** nanocomposite hydrogels, bone repair, regenerative medicine, 3D printing, stem cells

## Abstract

Hydrogels are three-dimensional (3D) water-swellable polymeric matrices that are used extensively in tissue engineering and drug delivery. Hydrogels can be conformed into any desirable shape using 3D bio-printing, making them suitable for personalized treatment. Among the different 3D bio-printing techniques, digital light processing (DLP)-based printing offers the advantage of quickly fabricating high resolution structures, reducing the chances of cell damage during the printing process. Here, we have used DLP to 3D bio-print biocompatible gelatin methacrylate (GelMA) scaffolds intended for bone repair. GelMA is biocompatible, biodegradable, has integrin binding motifs that promote cell adhesion, and can be crosslinked easily to form hydrogels. However, GelMA on its own is incapable of promoting bone repair and must be supplemented with pharmaceutical molecules or growth factors, which can be toxic or expensive. To overcome this limitation, we introduced zinc-based metal-organic framework (MOF) nanoparticles into GelMA that can promote osteogenic differentiation, providing safer and more affordable alternatives to traditional methods. Incorporation of this nanoparticle into GelMA hydrogel has demonstrated significant improvement across multiple aspects, including bio-printability, and favorable mechanical properties (showing a significant increase in the compressive modulus from 52.14 ± 19.42 kPa to 128.13 ± 19.46 kPa with the addition of ZIF-8 nanoparticles). The designed nanocomposite hydrogels can also sustain drug (vancomycin) release (maximum 87.52 ± 1.6% cumulative amount) and exhibit a remarkable ability to differentiate human adipose-derived mesenchymal stem cells toward the osteogenic lineage. Furthermore, the formulated MOF-integrated nanocomposite hydrogel offers the unique capability to coat metallic implants intended for bone healing. Overall, the remarkable printability and coating ability displayed by the nanocomposite hydrogel presents itself as a promising candidate for drug delivery, cell delivery and bone tissue engineering applications.

## 1. Introduction

Bones are composite materials with the extracellular matrix (ECM) consisting of minerals, water, collagen, proteins and lipids, protecting various vital organs and systems through their solid support and rigidness [1]. ECM contains a small number of type I collagen fibres, which is the organic part of ECM. The rest of the ECM contains inorganic matter from the mineral phase, mostly comprising hydroxyapatites [1,2]. Primary cells in bone tissues (osteoblasts, osteocytes and osteoclasts) actively communicate with each other to heal defects and facilitate bone growth, maintenance and resorption [3]. However, once a defect reaches a size and severity beyond the ability of bone to self-heal, an extraneous intervention must be applied to ensure healthy bone regeneration [4,5]. Bone defects usually range in severity, size of deformation, and root of causation. Causes include bone injuries, congenital and hereditary disorders, infections, and diseases [1,4,6]. Such defects can influence the healthy functioning and structural integrity of bones and joints and may consequently harm the wellbeing of individuals living with impairments. Clinically existing strategies for treating bone defects include autografts, allografts, alloplastic bone grafts, and metallic implants [1,3]. To date, bone defect treatments are one of the most common regenerative procedures, with more than 2 million bone grafts (83% autografts, 17% allografts and synthetic grafts) and 42 million metallic implant surgeries [7,8]. This substantial volume of procedures significantly impacts the healthcare system worldwide [7,8]. As such, developing effective treatment strategies is essential to reduce the enormous burden imposed on the healthcare system.

Human-derived allogenic bone grafts mimic biological properties, expediting recovery [9]. However, autografts and allografts pose risks, like donor-site pain, blood loss, disease transmission, and infections [9]. Alloplastic bone grafts, which blend synthetic and natural materials, offer advantages such as reduced disease transmission and easy sterilization. but often underperform due to implant engineering and fixation issues [10,11]. To overcome these limitations, innovative platforms like polymeric hydrogel scaffolds are being explored in regenerative medicine.

Polymeric hydrogels are 3D structures composed of hydrophilic polymer chains that can maintain their structures upon swelling due to crosslinking within the structure [12,13,14]. These 3D networks possess high water contents and a structure resembling that of ECM, which provides an ideal environment for cellular growth and tissue regeneration [12,15]. Polymeric hydrogels can be tailored to obtain the desired geometry, porosity, flexibility, and degradation rate, which helps them to integrate with host tissues due to their adjustable physiochemical properties [1,16]. The advantage of hydrogels, in contrast to traditional therapeutic techniques (such as allografts), include minimizing the risk of adverse immune responses [1,12,17]. This can be attributed to their biocompatible and biodegradable properties, with physiochemical characteristics resembling that of living tissues [1,18,19,20]. Gelatin methacryloyl (GelMA), as a polymerical hydrogel, is a gelatin derivative from denatured collagen, with methacrylic anhydride functional groups [12], and has attracted great attention in biomedical fields because of its bio-compatibility, biodegradability, bio-functionality, and ability to be photo-crosslinked [21,22]. GelMA-based scaffolds closely resemble the essential properties of the ECM, with interconnected macropores that facilitate cell adhesion, migration, growth, and proliferation [5,12,23,24]. However, traditional polymeric hydrogels (such as chitosan, alginate) are currently limited by their low mechanical strength that leads to premature failure and lack of biological functionalities needed for effective bone healing. To overcome these challenges, nanoparticles can be integrated in such polymeric systems, offering a promising solution [1,18,20,25]. The low strength, stiffness and toughness of GelMA hydrogel limit their applicability as implants because of their poor cell support [26]. Nanoparticles have the potential to enhance the mechanical strength and toughness of hydrogels, forming the development of either an intragranular or an intergranular structure framework [27,28].

Mineral-based nanoparticles, such as hydroxyapatite, bio-glass, calcium phosphate, and nanosilicates, have been widely used in bone regeneration application because of their inherent osteogenic properties [29]. Mineral-based nanomaterials have been shown to release favorable ions (such as Na^+^, K^+^, Mg^2+^, Sr^2+^, and Zn^2+^) that can regulate the activation of osteogenic cells to facilitate bone regeneration [29,30]. Among the different ions, zinc plays a crucial role in various biological processes, including bone metabolism and regeneration. Zinc is also involved in bone mineralization, collagen synthesis, and bone cell proliferation and differentiation [31]. Moreover, it is involved in the synthesis of bone matrix proteins, such as osteocalcin and collagen, which are essential for bone formation and mineralization [31,32]. However, mineral-based nanomaterials contain traces of elements, such as fluoride ions (F^−^) and hydroxyl ions (OH^−^), which cause an increase in polycrystalline structures, thereby reducing the diffusion of nutrients and inhibiting cell growth [30,33]. Additionally, the presence of excessive ions may lead to chronic inflammatory responses, and inhibit cell growth [30]. These drawbacks can be effectively addressed by sustaining the release of ions using MOFs. MOFs possess the capability to integrate osteogenic ions within their core structures, facilitating a controlled and gradual release of these ions [34]. MOFs are organic–inorganic hybrid porous nanomaterials composed of a regular arrangement of positively charged ions surrounded by organic ‘linker’ molecules, forming cage-like structures with large surface areas [34]. MOFs have several advantages, including low toxicity, bio-compatibility, and tunable shape, and have emerged as a promising carrier for delivering therapeutic molecules [34,35].

**Figure 1:**
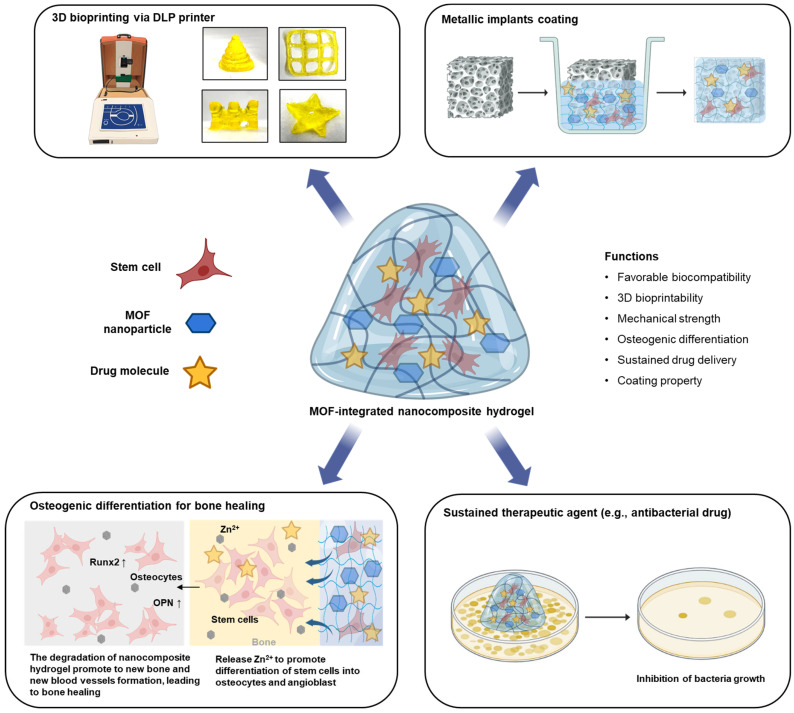
Illustration displays a ZIF-8-integrated nanocomposite hydrogel with versatile applications in bone repair, serving as both 3D bio-printable implants and coatings for inert metallic implants. It significantly promotes osteogenic differentiation in human stem cells and can sustain antibiotic drugs, exhibiting improved biological, and physicochemical properties.

Considering these advantages, herein, we have integrated zinc-based zeolitic imidazolate framework-8 (ZIF-8) nanoparticles, which belong to the class of MOFs, into GelMA polymer to form osteogenic hydrogel scaffolds, which can be used to treat non-load-bearing bone defects. The designed GelMA/ZIF-8 formulations were photo-crosslinked with the photo-initiator lithium phenyl-2,4,6-trimethylbenzoylphosphinate (LAP) using visible light at 405 nm. Furthermore, since the architecture of every bone defect is unique and patient-driven, we used Digital Light Processing (DLP)-based 3D bio-printing technology to synthesize the scaffolds. DLP was used because of its ability to quickly print high resolution structures. Our robust GelMA/ZIF-8 hydrogels were able to promote differentiation of human adipose-derived mesenchymal stem cells in vitro. Furthermore, we were also able to sustain the release of a model antimicrobial drug, vancomycin, using the GelMA/ZIF-8 hydrogels. Finally, the developed nanocomposite formulation was used as coating agent for the purpose of bio-functionalizing titanium-based metallic orthopedic implants intended for bone repair. Figure 1 highlights the various applications of our designed nanocomposite hydrogel.

## 2. Results and Discussion

### 2.1. Physicochemical Characterization Confirms the Successful Synthesis of ZIF-8 Nanoparticles

**Figure 2:**
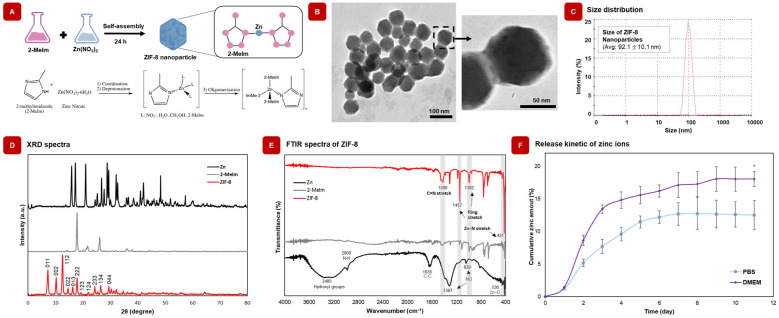
Characterization and fabrication of ZIF-8 nanoparticles and GelMA/ZIF-8 nanocomposite hydrogels. (**A**) Reaction scheme of the synthesis of ZIF-8. (**B**) TEM images showing size, morphology, and uniform distribution of ZIF-8 nanoparticles. (**C**) DLS data showing the size distribution of the ZIF-8 nanoparticles in ethanol solution. (**D**) XRD pattern of Zn, 2-Melm, and ZIF-8. The XRD analysis of ZIF-8 indicates high crystallinity of the prepared ZIF-8. (**E**) FT-IR spectra of ZIF-8, 2-methylimidazole (2-Melm), and zinc nitrate hexahydrate confirmed that zinc ions combined chemically with nitrogen atoms of the methylimidazole groups to form the imidazolate in a ZIF-8 product. (**F**) Cumulative release profile of zinc amount from the ZIF-8 nanoparticles. Results are indicated as mean ± S.D., (n = 5) * = *p* < 0.05.

The ZIF-8 nanoparticles were prepared according to the proposed reaction scheme by Ozterk et al. [36] (Figure 2A). The linker coordination of Zn^2+^ occurs at first, which is followed by the deprotonation of the 2-methylimidazole linker. Then, the monomers are oligomerized by linking together finite Zn^2+^ centres via the deprotonated 2-methyl imidazole ligands [36,37]. The TEM images of the synthesized ZIF-8 nanoparticles showed the morphology, size, and uniform distribution of the nanoparticles (Figure 2B). From the TEM, ZIF-8 showed a resemblance to a dodecahedron shape, which corresponds to the literature [36,37]. Through the use of DLS, the size of the ZIF-8 nanoparticles was determined to be 92.1 ± 10.1 nm (Figure 2C). Additionally, the ζ potential of the ZIF-8 nanoparticles was determined to be 29.5 ± 7.2 mV, which may help with charge interaction with GelMA. Next, the nanoparticles were chemically characterized to further validate the formation of ZIF-8 nanoparticles. From the ultraviolet-visible (UV-Vis) spectra graphs (Figure S1A), it was seen that there was an increase in absorbance of UV with increasing nanoparticle concentration, with the prominent absorbance peak of the nanoparticles at 215 nm. The XRD pattern of ZIF-8 nanoparticles (Figure 2D) revealed peaks corresponding to (011), (002), (112), (022), (013), (222), (114), (233) and (231), which match with previous findings and confirms the successful synthesis of the nanoparticles [38]. The FTIR spectra of ZIF-8 nanoparticles and their base components (Zinc and 2-methylimidazole) are shown in Figure 2E. The band 1590 cm^−1^ arises from C=N stretching, while 1457 and 1382 cm^−1^ correspond to the ring stretching of the ZIF-8. The Zn-N stretching vibration band at 421 cm^−1^ was also observed, which demonstrates successful synthesis of ZIF-8 nanoparticles.

### 2.2. ZIF-8 Nanoparticles Release Zn^2+^ Ions in Physiological Solutions

ZIF-8 nanoparticles degrade into 2-methlyimidazoles and Zn^2+^ in cell culture media and most common buffers due to the presence of various components [39,40,41], such as albumin, inorganic anions, and ascorbic acid that have a significant effect on the stability of ZIF-8 (Figure S1B). Due to the sustained release of Zn^2+^, which plays a key role in osteogenic properties, ZIF-8 can be used in bone healing applications [42]. Thus, understanding the degradation behavior of ZIF-8 under physiological conditions is essential to control the release of Zn^2+^ amount for their potential use in tissue regeneration. The residues of proteins and inorganic molecules can bind to Zn^2+^ ions and transport them into the cells to promote bone tissue regeneration [40]. DMEM and PBS were used to investigate the degradation behaviour of ZIF-8 nanoparticles as they are the most commonly used cell culture medium and buffer [43]. To determine the release kinetics of Zn^2+^ degradation, a linear curve was plotted between the Au and the concentration of Zn^2+^ ions, which was found using the zincon colorimetric assay, as shown in Figure S1C. Figure 2F displays the release profiles of Zn^2+^ ions from ZIF-8 nanoparticles. Equilibrium release was achieved after 11 days, corresponding to 12.48 ± 2.24% (in PBS) and 18 ± 1.16% (in DMEM). These results indicated that the presence of serum and various enzymatic activities in DMEM, which is similar to the physiological conditions, could induce degradation of ZIF-8, leading to effective bone repair. ZIF-8 nanoparticles were then integrated into GelMA to prepare nanocomposite hydrogels for bone repair.

### 2.3. Composition of GelMA Plays a Key Role on the Printability of GelMA/ZIF-8 Hydrogels

The composition of the ZIF-8-based nanocomposite hydrogel is critical to ensure the shape fidelity of the printed structure. Not all formulations are capable of bio-printing, which makes it important to optimize the formulation by 3D-bio-printing. Here, by 3D-bio-printing various GelMA hydrogel compositions using the Lumen X^TM^ DLP Bio-printer at fixed parameters (8 s exposure time, 80% power intensity), an optimal composition of components was determined. As illustrated in Figure S2A, only hydrogels printed with 15% *w*/*v* GelMA were printable, while hydrogels with 5% *w*/*v* and 10% *w*/*v* GelMA were not. The overall printability of hydrogels was determined by comparing the dimensions of the printed hydrogels to that of the original computer-aided design (4 mm diameter, 2 mm height) created with OnShape (PTC, Boston, MA, USA). All hydrogels at 15% *w*/*v* GelMA were printed to the desired dimensions (4 mm diameter, 2 mm height), with good shape retention and structural integrity (Figure S2B,D,E), whereas unprintable hydrogels did not meet the dimensions of the desired structure and lacked shape retention upon removal from the build platform (Figure S2C). Printability of hydrogels was not influenced by the concentration of ZIF-8 (Figure or results?). The observed trend with printability can be directly correlated to the concentration of GelMA instead. Upon exposure to visible light, the hydrogel compositions with smaller concentrations of GelMA did not print because they did not undergo substantial crosslinking [44,45]. Increased crosslinking occurs with higher concentrations of GelMA, creating a more rigid structure that is likely to maintain its configuration [44]. Increased crosslinking density limits the possibility of hydrogel deformation [44]. Next, we optimized different bio-printing parameters to effectively fabricate the GelMA/ZIF-8 nanoparticles.

### 2.4. Exposure Time and Power Have a Profound Effect on Shape Retention of 3D Printed GelMA/ZIF-8 Hydrogels

After determining the optimal GelMA/ZIF-8 composition for printing, we examined the effect of various printing parameters on the printability of the material. Three distinct power intensities, falling within the 10–30 mW/cm^2^ range, were examined at three specific levels: 70%, 80%, and 90%. These power settings were tested across various exposure durations, encompassing 5, 8, 10, and 15 s. It is noteworthy that after a 5-s exposure, the hydrogel printing process proved ineffective, irrespective of the chosen power intensity. (Figure S3A). At all remaining power intensities and exposure times, the hydrogels printed effectively. As illustrated in Figure S3B, there was variability in height observed for each printed hydrogel when considering the designated exposure times and intensities. At a higher power intensity (90%), there was less variation of height measurements at 8, 10, and 15 s, suggesting that hydrogels are sufficiently crosslinked at these parameters, so as to achieve the shape and dimensions specified by the intended stereolithography (STL) file. In contrast, Figure S3C showed no variation in diameter at different power intensities or exposure times. This may be due to sufficient crosslinking having a more profound impact on achieving a set height as compared to its ability to achieve a set diameter. To conclude, 15% GelMA is needed for bio-printing, along with 1% LAP and 0.1% Tartrazine. Moreover, 80% power and 8 s of light exposure are sufficient to achieve shape retention of the fabricated GelMA/ZIF-8 hydrogels. In the subsequent sections, we use these optimized parameters to characterize the designed hydrogels.

### 2.5. Integration of ZIF-8 Nanoparticles in GelMA Alters the Physicochemical Behavior of the Pristine Hydrogel

#### 2.5.1. GelMA/ZIF-8 Hydrogels Display Lower Swelling Ratio When Compared to GelMA Hydrogels

**Figure 3:**
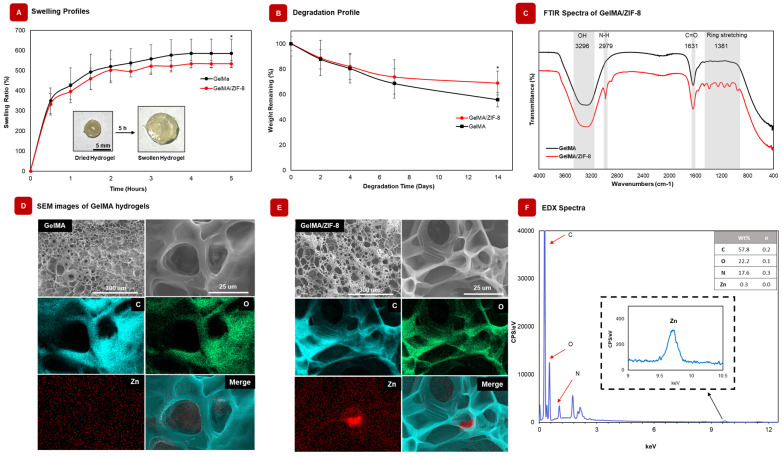
Physiochemical Characterization of GelMA/ZIF-8 nanocomposite hydrogels. (**A**) Swelling ratio of GelMA and GelMA/ZIF-8 (3 mg/mL) nanocomposite hydrogels (n = 5). Incorporation of ZIF-8 resulted in a reduced swelling rate of the hydrogels. (**B**) Percent degradation of GelMA and GelMA/ZIF-8 (3 mg/mL) nanocomposite hydrogels (n = 5). ZIF-8 reduces the rate of degradation of the hydrogels after 14 days. (**C**) FTIR analysis of GelMA, ZIF-8, and GelMA/ZIF-8. (**D**) SEM image and elemental mapping images of GelMA and (**E**) GelMA/ZIF-8 hydrogel. Both hydrogels showed similar porosity. As revealed by elemental mapping, two major components (C, O elements) of GelMA hydrogels were present in both the groups whereas, Zn was only found in GelMA/ZIF-8 hydrogels. These results indicate that ZIF-8 was successfully incorporated in GelMA hydrogels. (**F**) EDX analysis of GelMA/ZIF-8 further verifying the presence of Zn in the nanocomposite hydrogel. * = *p* < 0.05.

Swelling ratio is a critical physical property of hydrogels intended for use in bone repair. A high rate of swelling in implanted hydrogels can be detrimental to the well-being of the neighboring tissue [46]. Figure 3A demonstrates the effect of ZIF-8 nanoparticles on the swelling behavior of GelMA hydrogels. At equilibrium, GelMA hydrogels displayed a swelling ratio of 586 ± 71.51%, whereas GelMA/ZIF-8 nanocomposite hydrogels swelled to 534 ± 16.71%. As such, integration of ZIF-8 nanoparticles can reduce the rate of swelling and improve the applicability of the hydrogels for treating non-load-bearing bone defects.

#### 2.5.2. GelMA/ZIF-8 Hydrogels Degraded Slower than GelMA Hydrogels

The rate of degradation of hydrogels is crucial for the release of zinc ions or encapsulated therapeutics intended for bone repair or fighting infection. However, a rapid rate of degradation leads to implant failure because of its inability to support cells of adhesion [47,48]. Therefore, it is of paramount interest to the researchers to evaluate the rate of degradation of such bio-based implants. To study the influence of ZIF-8 on hydrogel degradation, the percent degradation was studied over 2 weeks in PBS with 1% p/s at 37.5 °C. Findings suggest that the presence of ZIF-8 slowed the rate of degradation. On day 14, there was significant reduction in degradation between GelMA/ZIF-8 and GelMA hydrogels, where the weight remaining was 68.9 ± 9.40% and 55.8 ± 7.79%, respectively (Figure 3B). As suggested by Liu et al. [49], ZIF-8 nanoparticles used in hydrogel structures may strengthen the mechanical properties of hydrogels, which in turn can reduce their rate of degradation. A reduced rate of degradation is beneficial for using these hydrogels as implants for bone repair.

#### 2.5.3. Presence of ZIF-8 in GelMA/ZIF-8 Hydrogel Was Confirmed Using FTIR Analysis

FTIR spectra were used to validate the presence of ZIF-8 in GelMA/ZIF-8 hydrogels. Figure 3C displays the band characteristic of ZIF-8 wat 2979 and 1381 cm^−1^, which represents the N-H stretching of the imidazole ring and the bending of the ring, respectively [50]. The band characteristic of GelMA is also present at 3296 and 1631 cm^−1^, which can be attributed to O-H stretching and amide bonds (with C=O stretching vibrations), respectively [50]. GelMA/ZIF-8 hydrogels exhibited comparable peak patterns, including N-H stretch and ring stretching, reinforcing the existence of ZIF-8 within the nanocomposite group. Further chemical proof is obtained by using EDX spectra, discussed in Section 2.5.5.

#### 2.5.4. SEM Images and Elemental Mapping Demonstrated the Successful Integration of ZIF-8 in GelMA/ZIF-8 Hydrogel

The cross-sectional morphology of GelMA and GelMA/ZIF-8 hydrogels were investigated using SEM (Figure 3D,E). The pore size was observed to be similar for both GelMA and GelMA/ZIF-8 hydrogels. Elemental mapping revealed that major components for both samples included carbon and oxygen. Zinc was only found in GelMA/ZIF-8 hydrogels, which indicated that ZIF-8 was successfully incorporated in GelMA/ZIF-8 hydrogels.

#### 2.5.5. Presence of ZIF-8 in GelMA/ZIF-8 Hydrogels Was Further Confirmed with EDX Analysis

EDX analysis was performed to further understand the elemental composition of GelMA/ZIF-8 hydrogel. The major components in the sample included carbon, nitrogen, and oxygen. The minor component consisted of zinc, which highlighted the presence of ZIF-8 nanoparticles in the sample. The component with the highest weight percent was carbon at 57.8% and following was oxygen and nitrogen at 22.2% and 17.6%, respectively (Figure 3F). Zinc had a weight percent of 0.3%. Notably, zinc was not present in the GelMA group. Next, we use the designed nanocomposite formulation as a coating agent and characterize GelMA/ZIF-8-coated metallic implants using SEM, elemental mapping, and EDX.

### 2.6. Orthopedic Metallic Implants Can Be Coated with GelMA/ZIF-8 Hydrogels

**Figure 4:**
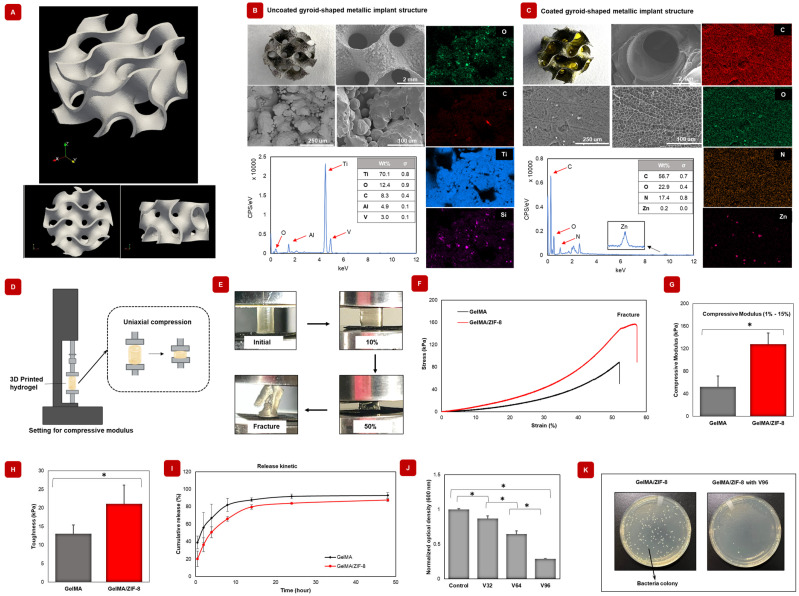
Application and physicochemical characterization of GelMA/ZIF-8 hydrogels. (**A**) Fabrication of the gyroid structure was carried out using laser-powder bed fusion (LPBF) at an additive manufacturing facility (Additive Design in Surgical Solutions, London, ON, Canada) using a commercial 3D metal printer (AM400, Renishaw plc, Wotton-under-Edge, Gloucestershire, UK). Printing parameters were: laser power 200–400 W, scanning speed 10,000–20,000 points/second, particle diameter 15–45 microns, spot size 70 microns. After consolidation, the test components were heat treated (24-h cycle of stress relief, 850 °C for 1 h then passive cool down) to reduce residual stress. (**B**) Photograph, SEM images, and EDX spectre of gyroid structure (**C**) Photograph, SEM images, and EDX spectre of gyroid structure coated with GelMA/ZIF-8 hydrogel. (**D**) Schematic design of mechanical testing on 3D printed hydrogel with a cylindrical dimension (3 mm × 6 mm). (**E**) Photographs revealing the notable compression resistance of nanocomposite hydrogel. (**F**) Compressive stress−strain curves. (**G**) The compression modulus of samples was determined in the range between 1 and 15 % of the stress-strain (n = 5). (**H**) Toughness of the samples (n = 5). *p* values were determined by a student *t*-test; * *p* ≤ 0.05. (**I**) Cumulative release profile of vancomycin drug in gels. (**J**) Evaluating in vitro antimicrobial activity at different concentrations of gels. (n = 3) V32, V64, and V96 indicate drug concentrations, vancomycin 32, 64, and 96 ug/mL, respectively. (**K**) Images of bacterial cultured plates shows decreased bacterial populations in drug-loaded GelMA/ZIF-8 hydrogel.

The gyroid structure was fabricated utilizing laser-powder bed fusion (LPBF) within an additive manufacturing facility (Figure 4A). The morphology of GelMA and GelMA/ZIF-8 hydrogel-coated gyroid-shaped metallic implants were investigated with SEM. Figure 4B,C show SEM images of GelMA-coated and nanocomposite hydrogel coated gyroid-shaped metallic implants, respectively. The uncoated gyroid structure showed irregular spheres on its surface, as confirmed by SEM imaging. In contrast, the gyroid coated with nanocomposite hydrogel had a porous hydrogel surface. Elemental mapping revealed that the uncoated gyroid-shaped metallic implants consisted mainly of Ti. Conversely, the hydrogel-coated gyroid showed the presence of carbon, oxygen, nitrogen, and zinc, which are the primary components of the hydrogel.

### 2.7. GelMA/ZIF-8 Hydrogels Displayed Superior Mechanical Strength and Toughness When Compared to GelMA Hydrogels

Several nanoparticles, such as carbon nanotube [51] and graphene oxide [52], have been shown to enhance the mechanical properties of GelMA hydrogels [53]. As such, ZIF-8 nanoparticles may also serve as a potential aid to improve the mechanical properties of GelMA hydrogels. To determine the effect of ZIF-8 nanoparticles on the mechanical strength and toughness of the GelMA hydrogel, compressive modulus, strength, and toughness were measured using static compression loading. Figure 4D illustrates the set-up of the compression tests. Photographs in Figure 4E illustrate the compressibility of GelMA/ZIF-8 hydrogels. The stress–strain curves shown in Figure 4F are obtained using the compression test. Figure 4G shows that a significant increase in the compressive modulus from 52.14  ±  19.42 kPa to 128.13  ±  19.46 kPa was obtained when ZIF-8 nanoparticles were added to GelMA hydrogels (n = 5). Next, the toughness of hydrogels was also determined from the area below the stress–strain curve until fracture (Figure 4H). An increased toughness was observed in GelMA/ZIF-8 hydrogels from 13.02 ± 2.39 kPa to 21.14 ± 5.06, with a significant difference between the samples (n = 5). These findings demonstrate that the incorporation of ZIF-8 can effectively enhance the mechanical strength and toughness of the hydrogel. Improved mechanical properties may also be a reason for the slower rate of degradation (as observed in Section 2.5.2). It is speculated that the molecular interactions between the positively charged ZIF-8 nanoparticles and the polymer chains may help support and enhance the mechanical properties of the nanocomposite hydrogels. This improvement of material stiffness may, in turn, support sufficient cellular growth and integration [54,55].

### 2.8. GelMA/ZIF-8 Hydrogels Sustain the Release of Model Antibiotic Vancomycin at Physiological Conditions

Effective post-transplant treatment requires minimizing the risk of infection. However, administering high doses of systemic antibiotics can cause potential systemic toxicity, leading to treatment failure and unwanted side effects [56]. To address this issue, a promising approach is to use polymeric delivery vehicles, which can offer appreciable concentrations at the target site while causing fewer side effects than systemic administration [57,58]. For drug delivery applications, polymeric hydrogels are frequently used due to their (a) high porosity and water retention, which is suitable for hydrophilic drug encapsulation, (b) reproducible drug loading, and (c) sustained drug release profiles [59]. In this study, the hydrophilic vancomycin was used as a model antimicrobial drug to investigate its cumulative release from GelMA/ZIF-8 hydrogels. Vancomycin has a half-life of 4 to 6 h, and as such, a sustained release is desirable for improved therapeutic efficacy. Here, 96 ug/mL of vancomycin was loaded into dried hydrogels and the release was studied using UV-Vis spectroscopy. Both GelMA (92.97 ± 3.1%) and GelMA/ZIF-8 (87.52 ± 1.6%) were able to sustain the release of vancomycin over 48 h (Figure 4I). Next, we assessed the antimicrobial efficacy of three different drug concentrations by exposing the drug-loaded nanocomposite hydrogels to *E. coli* for a period of 24 h. All three concentrations showed significant progressive bacterial reduction compared to the control (Figure 4J). Figure 4K shows images of bacterial plates, which confirmed that drug-loaded GelMA/ZIF-8 hydrogel has effective antibacterial properties.

### 2.9. Antioxidant Agent-Loaded Nanocomposite Hydrogels Can Scavenge Free Radicals

Antioxidant agents play a vital role in maintaining cellular health by neutralizing harmful free radicals. Nanocomposite hydrogels can be used to deliver and release these molecules [60]. To demonstrate the scavenging activity of the designed ZIF-8 integrated nanocomposite hydrogel, the antioxidant agent ascorbic was loaded in the lyophilized scaffold. The activity of the loaded antioxidant agents can be evaluated using the 2,2-diphenyl-1-picrylhydrazyl (DPPH) assay, a method where the released antioxidant species can react with the stable free radical. Figure S4 shows an increase in the scavenging activity of the ascorbic acid-loaded nanocomposite hydrogel with an increase in the concentration of DPPH. Such robust scavenging activity demonstrates the ability of the designed GelMA/ZIF-8 hydrogel to serve as a carrier of antioxidant-based therapeutics.

### 2.10. GelMA/ZIF-8 Hydrogels Show Excellent Cyto-Compatibility and Osteogenic Differentiation Abilities

#### 2.10.1. In Vitro Cyto-Compatibility of Mineral-Based Nanocomposite Hydrogels

**Figure 5:**
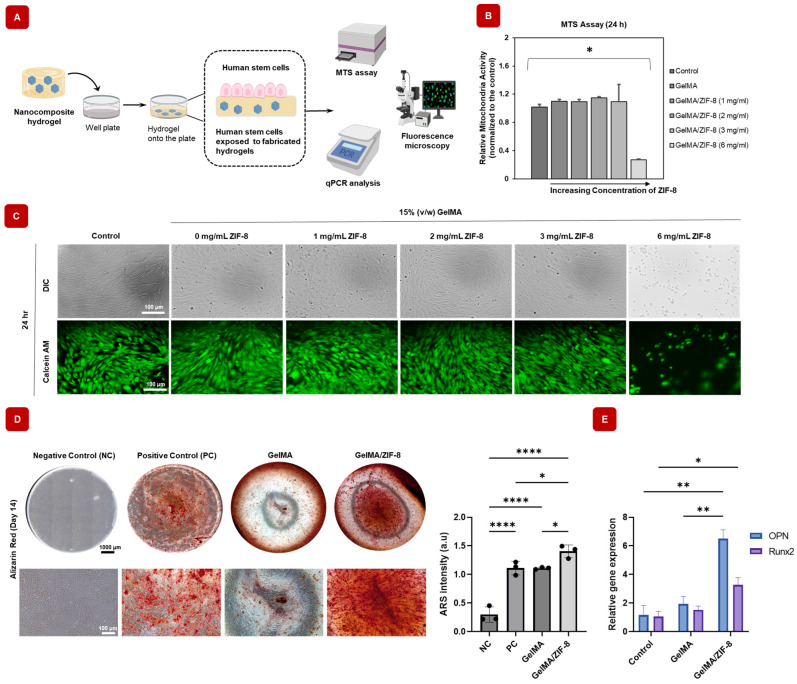
In vitro cyto-compatibility and osteogenic differentiation potential of GelMA/ZIF-8 hydrogels with 2D human stem cell culture. (**A**) Schematic visualization of the method by which the cells were exposed to the hydrogels. (**B**) MTS cell proliferation assay showing no adverse effect on stem cells when ZIF-8 is equal to or less than 3 mg/mL in the nanocomposite hydrogels (n = 3). (**B**) These were determined using a two-tailed *t*-test. A *p*-value 0.05 indicates statistical significance, which was displayed as * *p* < 0.05. (**C**) DIC and fluorescence images of stem cells with various nanocomposite hydrogel concentrations. Scale bar indicates 100 μm. (**D**) Images of alizarin red stain show significantly higher production of mineralized deposition in hASCs treated with GelMA/ZIF-8 hydrogel compared to Negative control (no dexamethasone in media) or Positive control (with dexamethasone in media). Incorporation of ZIF-8 nanoparticles resulted in a substantial improvement in the production of the mineralized matrix in presence of osteogenic media, as determined by quantifying the amount of ARS stained in the mineralized matrix. (**E**) Introduction of ZIF-8 nanoparticles also led to an upregulation of osteogenic markers, specifically OPN and RUNX2, corresponding to the gene expressions related to osteogenic differentiation of hASCs. Differentiation of the cells cultured in three groups (n = 3) for 21 days. * = *p*-value < 0.05, ** = *p*-value < 0.01, and **** = *p*-value < 0.0001.

When considering bio-medical applications of GelMA/ZIF-8 hydrogels, it is crucial that the components are cyto-compatible. GelMA is often sought after for tissue engineering and drug delivery applications because of its high bio-compatibility and low cytotoxicity [23,24,61]. Additionally, although ZIF-8 nanoparticles have bio-compatible properties, a threshold concentration must be examined using a stem cell proliferation assay to determine its compatibility with biological tissue [62]. Here, cyto-compatibility was confirmed by seeding hASCs on GelMA/ZIF-8 nanocomposite hydrogels at varying concentrations of ZIF-8 (Figure 5A). Figure 5B indicates that there was no difference in cell proliferation between the control (hASCs), GelMA, and GelMA/ZIF-8 hydrogels up to 3 mg/mL. The morphology of cells was imaged by bright field microscopy with different concentrations of nanocomposite hydrogels. Furthermore, fluorescence microscopy with Calcein AM staining was performed to assess the effect of GelMA/ZIF-8 hydrogels on the shape and morphology of the hASCs (Figure 5C). Both phase contrast and fluorescence microscopy showed that GelMA/ZIF-8 hydrogels did not have any detrimental effects on hASCs up to 3 mg/mL of ZIF-8 concentration. However, with GelMA/ZIF-8 at 6 mg/mL, there was a significant difference, with a distinct reduction in cell proliferation. Similar assays from Hoop et al. suggest that the cytotoxicity of ZIF-8 at higher concentrations results from increased release of Zn^2+^, which impacts the mitochondrial reactive oxygen species production [62]. Combining the results of cell proliferation studies, 3 mg/mL ZIF-8 nanoparticles is the advisable concentration to fabricate GelMA/ZIF-8 hydrogels.

#### 2.10.2. GelMA/ZIF-8 Hydrogels Can Differentiate hASCs toward Osteogenic Lineage

Mineral-based ions are essential dietary requirement and are involved with several biological functions [63]. In particular, zinc performs multiple functions related to the immune system, cell division, fertility, and cell growth and maintenance [64]. In bone biology, zinc is proven to be an important element for the formation of bone, mineralization, development, and maintenance of healthy tissue [64]. In this regard, zinc has widely been used along with other bio-materials, such as bioactive glasses for bone regeneration applications [64,65]. Given the role for zinc in osteogenic differentiation [31], we assessed the osteogenic activities of zinc in the form of ZIF-8 nanoparticles contained in GelMA/ZIF-8 hydrogels. To analyze the formation of mineral depositions, a crucial indicator of late-stage osteogenesis, an Alizarin Red S (ARS) assay, was performed on day 14. Encouragingly, the introduction of GelMA/ZIF-8 nanocomposite hydrogel significantly enhanced matrix mineralization in hASCs when compared to the group without nanoparticles (Figure 5D). To determine the osteogenic activities at the genetic level, RT-qPCR was performed. First, hASCs were seeded in 24 well plates at a cell density of 0.05 × 10^6^ per well. Then, 3D-printed GelMA and GelMA/ZIF-8 hydrogels were co-cultured (n = 3). After 21 days, osteogenic gene levels of OPN and RUNX2, which are common osteogenic bone markers, were determined as shown in Figure 5E. Significantly higher levels of OPN and RUNX2 expression can be seen in GelMA/ZIF-8 hydrogels compared to the GelMA and the blank groups. These results demonstrated that the delivery of zinc using GelMA/ZIF-8 hydrogels can promote effective osteogenic differentiation, which is vital for bone repair applications.

#### 2.10.3. Complex Shapes of GelMA/ZIF-8 Hydrogels Can Be Fabricated Using DLP-Based 3D Bio-Printing

**Figure 6:**
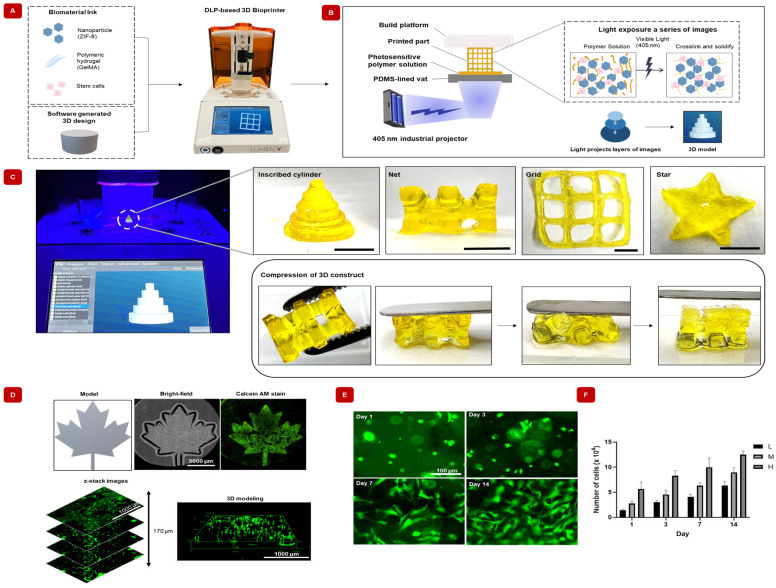
Feasibility and applicability of DLP-based 3D bio-printing of GelMA/ZIF-8 hydrogels using various CAD-designed models. (**A**) Illustration of the strategy implemented in the fabrication of 3D structures using a DLP-based 3D bio-printer. (**B**) Graphic displaying the principle of digital light processing (DLP)-based 3D bio-printing technology. The light-sensitive liquid polymer solution is placed in a vat. A visible blue-light (405 nm) projector exposes a series of images onto the polymer solution. The areas of polymer solution that are exposed to the light crosslink and solidify into a single layer. The build platform moves up to allow each layer to stack and build the model. (**C**) Photographs displaying various 3D printed nanocomposite hydrogels scaffolds. Scale bar indicates 5 mm. Optical images showing elasticity of the 3D printed nanocomposite hydrogels after deformation. (**D**) Images of designed model and 3D bio-printed structure showing a bright-field microscopic image and fluorescent image of live cells. (**E**) Fluorescent images of live cells were determined at different time points. (**F**) The proliferation of cells over time is influenced by variations in cell seeding density. (Initial cell seeding number per well indicated as L: 0.5 × 10^4^, M: 1 × 10^4^, and H: 2 × 10^4^).

3D bio-printing enables the fabrication of complex structures with high precision and reproducibility. To achieve this, bio-material inks with suitable printability and bio-compatibility are necessary. In this study, we investigated formulations containing GelMA, ZIF-8, and hASCs as bio-inks for 3D bio-printing (Figure 6A). Figure 6B shows the principle of the DLP-based bio-printer. Briefly, the first step requires the creation of a 3D model of the object that needs to be printed. This can be carried out using computer-aided design (CAD) software, such as OnShape. The 3D model is sliced into multiple 2D layers that are limited only by the resolution of the printer being used. A vat of liquid bioink is placed above a DLP projector, which projects a pattern of light onto the bio-ink. The pattern of light corresponds to the 2D image sliced from the 3D model, and the areas of the bio-ink that are exposed to this patterned light crosslink to form the final object. The process is repeated for each 2D layer, building the object up finally in a layer-by-layer format. Figure 6C shows images of the nanocomposite GelMA/ZIF-8 hydrogels printed with various 3D models along with their compressibility. Figure 6D shows bio-printed GelMA/ZIF-8 hydrogels encapsulated with hASCs with the Calcein AM stained cells, showing the presence of healthy cells along the thickness of the GelMA/ZIF-8 hydrogel. Furthermore, the bio-printed cells exhibited a noticeable increase in proliferation over time (Figure 6E) with very few dead cells (Figure S5), demonstrating the ability of the designed nanocomposite hydrogels to support and maintain cells for effective bone repair.

## 3. Conclusions

In conclusion, this study proves the printability of GelMA/ZIF-8 hydrogels developed for bone regeneration. It was determined that, to effectively 3D print a structure, a concentration of 15% GelMA is required along with a minimum of an 8 s exposure time at 80% power intensity. Moreover, the designed nanocomposite formulation can also be used as a coating agent for orthopedic metallic implants.

Chemical characterization using FTIR and EDX analysis proved the presence of ZIF-8 in the hydrogel. Furthermore, the ZIF-8 nanoparticles reduced the hydrogel’s swelling ratio, which is beneficial when used as an implant for treating non-load-bearing defects (e.g., the calvarial defect). The presence of ZIF-8 nanoparticles in hydrogels also reduced the rate of the degradation significantly, which may prolong cell support and infiltration in vivo. This relatively low degradation rate should be exploited in vivo to evaluate the effect of the nanocomposite hydrogel on therapeutic potential.

Additionally, ZIF-8 nanoparticles enhanced the mechanical strength and toughness of the hydrogels, which could help prevent the premature degradation of the scaffolds. In vitro cyto-compatibility of nanocomposite hydrogels revealed that there was a threshold concentration of 3 mg/mL up to which ZIF-8 exhibits cyto-compatible characteristics. Lastly, the incorporation of ZIF-8 nanoparticles significantly improved the osteogenic properties of the nanocomposite hydrogels in vitro. This enhancement was observed through the up-regulation of key osteogenic markers, namely *RUNX2* and *OPN*, which plays a crucial role in promoting the osteogenic differentiation of stem cells. Furthermore, the presence of ZIF-8 nanoparticles resulted in increased mineral depositions, further indicating the enhanced osteogenic potential of the nanocomposite hydrogels. Through extensive analysis, we demonstrate the remarkable potential of GelMA/ZIF-8 hydrogels in various bio-medical applications, such as drug delivery (examples of therapeutics include antimicrobial and antioxidant agents), 3D bio-printing, and coating metallic implants. We would also like to acknowledge that in vivo experiments must be conducted in future to further assess the bioactivity of our designed nanocomposite hydrogel. Taken together, this newly developed ZIF-8 nanoparticle reinforced hydrogel with its multifaceted biomedical properties including (i) controllable zinc ion release ability to facilitate bone repair, (ii) ability to deliver antibiotic drugs, (iii) superior bio-compatibility with respect to stem cell adhesion, proliferation, and differentiation, as well as (iv) 3D bio-printability of stem cells, using visible light-induced crosslinking, make them an attractive candidate for use in orthopedic implants and critical size bone defect therapy.

## 4. Materials and Methods

### 4.1. Preparation of GelMA Pregel Solution

GelMA was fabricated using established protocol [24]. In an Erlenmeyer flask, gelatin at 10% *w*/*v* in PBS was stirred with a magnetic stir bar for 1 h at 60 °C, until solubilized. After dissolution of gelatin, methacrylic anhydride (0.8 mL/g of gelatin) was slowly added dropwise to the polymer solution and was left to rotate for an additional 2 h at 60 °C. Next, the GelMA was diluted with heated PBS (100 mL) and the mixture was dialyzed for about 1 week by using a dialysis membrane (12–14 kDa molecular weight cut-off) with distilled water (50 °C). The water was changed twice daily, to remove the toxic unreacted methacrylic anhydride from the GelMA. The GelMA was stored in 50 mL-Falcons for 4 days at −80 °C and subsequently freeze-dried.

### 4.2. Preparation of ZIF-8 Nanoparticles

ZIF-8 was synthesized following the literature with some modifications [31]. First, 150 mg of zinc nitrate hexahydrate was solubilized in 5 mL of DI water. Next, 330 mg of 2-methylimidazole was solubilized in 100 mL of methanol. Then, the two solutions were combined and stirred overnight (12 h) at room temperature. The resultant solution was centrifuged at 14,000 rpm for 30 min. Upon separation of the components, the precipitated ZIF-8 nanoparticles were washed, re-suspended in methanol and centrifuged: this process was repeated once more to remove unreacted species. The final pellet was resuspended in ethanol and was dried for further use.

### 4.3. Electron Microscopy to Determine Morphology of ZIF-8 Nanoparticles and GelMA/ZIF-8 Hydrogels

To assess the topography of synthesized ZIF-8 nanoparticles, transmission electron microscopy (TEM) was performed using a Philips 420 transmission microscope. To characterize the morphology of synthesized GelMA and GelMA/ZIF-8 nanocomposite hydrogels, scanning electron microscopy (SEM) was performed using Hitachi SU8230 and images of the lyophilized (Labconco, Kansas, MO, USA) hydrogels were taken at an acceleration voltage of 20 kV. Energy-dispersive X-ray (EDX) spectroscopy and elemental mapping were also carried out using an X-Flash 6160 EDX detector (Bruker, ESPRIT analytical software, Santa Barbara, CA, USA) to investigate the major components of the samples.

### 4.4. Zeta Potential and Dynamic Light Scattering (DLS) Analysis of ZIF-8 Nanoparticles

To determine the zeta potential and size distribution of ZIF-8 nanoparticles, dynamic light scattering (DLS) was performed using Zetasizer Nano ZS instrumentation (Malvern Instruments, Malvern, UK) at 25 °C.

### 4.5. DLP-Based 3D Bio-Printing of GelMA/ZIF-8 Hydrogels

#### 4.5.1. Preparation of Bio-ink

The pre-gel solution called bio-ink was prepared as follows. First, lyophilized GelMA was solubilized in PBS (pH 7.4) to a 20% GelMA concentration in an oven at 70 °C. Next, ZIF-8 nanoparticles were weighed and suspended in a solution of ethanol to a concentration of 10 mg/mL. The solution was sonicated (505 Sonic Dismembrator, Fisher Scientific, Waltham, MA, USA) at 15% amplitude for 1 min to ensure homogenization. Table 1 highlights the amount of stock ZIF-8 that was dried in a 15 mL falcon tube per 300 µL of the GelMA hydrogel solution, to achieve the desired concentration of the ZIF-8 in the final scaffold. The ZIF-8 solution was dried in 15 mL falcon tubes overnight, while covered with parafilm with fine holes to allow for airflow, while minimizing contamination. Once dried, 300 µL of GelMA hydrogel solution was added to the falcon tube with dried ZIF-8 and sonicated (15% power, 30 s) to obtain the final GelMA/ZIF-8 nanocomposite hydrogel mixture.

GelMA/ZIF-8 nanocomposite hydrogel formulations were prepared with varying concentrations of ZIF-8 nanoparticles (0, 0.5, 1, 2, 3 mg/mL). Concentrations of the photo-initiator, LAP (1% *w*/*v*) and photo-absorber, tartrazine (0.1% *w*/*v*) were kept constant for every formulation. Table 2 illustrates the general scheme for preparation of the polymeric hydrogel depending on the concentration of GelMA. Upon successfully preparing individual components, GelMA, LAP, tartrazine, and DI water were added to 15 mL falcon tubes and vortexed to ensure all components were adequately mixed to create a homogenous mixture of the bio-ink. The finalized GelMA hydrogel formulation was kept in the oven at 50 °C until ready to be used for 3D bio-printing. For formulations containing cells, human derived adipose stem cells (hDASCs) were digested by 0.125% trypsin-EDTA and centrifuged for 2 min. The GelMA/ZIF-8 nanocomposite hydrogels mixture was added to hDASCs at a final density of approximately 2 × 10^6^ cells mL^−1^.

#### 4.5.2. 3D Bio-Printing of GelMA/ZIF-8 Hydrogels Using LumenXTM

The Lumen X^TM^ DLP Bio-printer (CELLINK) was used for fabricating the hydrogels. Lumen X^TM^ uses a visible light source at 405 nm and 10–30 mW/cm^2^ to crosslink polymers. Polydimethylsiloxane (PDMS) dishes (CELLINK) were used to 3D bio-print the hydrogels. The models for bio-printing were designed using an online computer-aided design (CAD) software called Onshape (Format: accessed on 1 May 2023, https://www.onshape.com/en/) and subsequently downloaded to LumenX^TM^. The printing process was initiated by calibrating the build plate of the printer first. Upon completion of calibration, bio-printing parameters were set to a fixed power of 80% (intensity range between 10–30 mW/cm^2^), exposure time of 8 s, and first layer time scale factor of 4X to allow extra time for the cured layer to gain contact with bio-printing dish and hydrogel. To print the 25 µL cylinder, 100 µL of the composed GelMA hydrogel was dispensed onto the Lumen X+^TM^–PDMS Dish. Bio-printing occurred in a dark room as to avoid visible light interreference. After the bio-printing was completed, the 3D hydrogel was removed from the build platform with a plastic razor. For shape fidelity confirmation and comparison, visual analysis of hydrogels was performed. The hydrogels were transferred to a blank background where both height and diameter were measured to ensure that the printed hydrogel aligned with set measurements of the fabricated structure created through OnShape. Through visual analysis, recorded dimensions, and observed structural integrity of the 3D printed hydrogel, an optimal GelMA hydrogel formulation was thus determined.

#### 4.5.3. Lyophilization of Hydrogels

GelMA/ZIF-8 nanocomposite hydrogel solution was pipetted in 96 well plates and crosslinked using visible light (405 nm) for 25 min to ensure sufficient crosslinking. The hydrogels were carefully removed from the well plate by using curved forceps, placed in a petri dish and covered with PBS and placed on a shaker for 12 h to remove unreacted components. The hydrogels were transferred to a container and frozen at −80 °C. Subsequently, the frozen hydrogels underwent lyophilization using a Labconco system, USA.

#### 4.5.4. Physical Characterization: Swelling

To compare swelling behaviour in the presence of ZIF−8 nanoparticles, dried 150 µL GelMA and GelMA/ZIF-8 hydrogels (n = 5) were prepared and weighed. Swelling behaviour was assessed by placing dried hydrogels in a 24-well plate and soaking in PBS (1 mL) at 37.5 °C. Equilibrium swelling was determined by weighing the swollen hydrogels at 30-min intervals for a total of 5 h. For each time point, the swelling ratio (%) was calculated using the following equation:

- Equation (1): Swelling Ratio (%)

(1) $$Swelling\;Ratio\left(\%\right)=\frac{{(W}_{s}-W_{o})}{W_{o}}*100$$

where W_S_ is the weight of the swollen hydrogel at specific time intervals and W_o_ is the initial weight of the dried hydrogel.

#### 4.5.5. Physical Characterization: Degradation

To compare degradation behaviour in the presence of ZIF-8 nanoparticles, dried 150 µL GelMA and GelMA/ZIF-8 hydrogels (n = 5) were prepared and weighed as the initial weight. The hydrogels were incubated in 24-well plates and were soaked in PBS (1 mL) with 1% p/s at 37.5 °C for 2 weeks. On different days, the hydrogels were dehydrated and weighed. The degradation rate (%) was calculated using the following equation:

- Equation (2): Degradation rate (%)

(2) $$Degradation\;rate\left(\%\right)=\frac{W_{o}-W_{D}}{W_{D}}*100$$

where W_o_ is the initial weight of the dried hydrogel and W_D_ is the weight of the dried hydrogel at specific time intervals.

#### 4.5.6. Chemical Characterization: Fourier Transformed Infrared (FTIR)

Fourier Transformed Infrared (FTIR) spectra were collected on a Nicolet Summit FTIR Spectrometer (Thermo Fisher, Norristown, PA, USA). All FTIR experiments were performed at room temperature and the spectra were recorded between 400 and 4000 cm^−1^.

#### 4.5.7. Mechanical Characterization of GelMA/ZIF-8 Hydrogels

Mechanical testing was performed using the Instron 3345 universal mechanical testing machine (Instron, Norwood, MA, USA) to determine the compressive modulus of the nanocomposite hydrogels and assessed under unconfined uniaxial compression with a 10 N load cell (n = 5). The analyzed samples were fabricated in a cylinder shape (diameter of 6 mm and height of 3 mm: total volume of ~50 uL per sample). All mechanical tests were performed on the swollen hydrogels and the diameter of samples was measured using a calliper prior to the test to ensure exact dimensions of the samples. The compression probes were covered with sandpaper to prevent the gel from sliding during the test. A compression rate of 0.30 mm/min was used. The compressive modulus was measured by fitting the linear region of the stress-strain slope, ranging between 1 and 15% strain. Samples were compressed until fracture was measured directly with the Instron 3345. The toughness was calculated from the area below the stress–strain curve until fracture. The calculation used in the following equation:

- Equation (3): Toughness (kPa)

(3) $$Toughness\;(kPa)=\int_{x_{0}}^{x_{f}}\sigma (x)dx$$

where $x_{0}$ corresponds to the starting point of the strain, $x_{f}$ corresponds to the fracture point of the strain, and σ corresponds to the starting point of the stress during compression.

### 4.6. Zinc Ion Release from ZIF-8 Nanoparticles

The Zn^2+^ release behaviour was performed by the zincon spectrophotometric (Sigma, Cat. No. 96440, St. Louis, MI, USA) method following the manufacturer’s protocol [66,67]. To monitor the released Zn^2+^ from 3 mg/mL of ZIF-8, nanoparticles (n = 5) were soaked in both PBS and DMEM with pH 7.4 at 37 °C. During the process, the leaching liquor was collected by centrifugation at 4000 rpm for 10 min and quantified by zincon spectrophotometry at 620 nm. The obtained standard curve was utilized to calculate the cumulative release quantity of Zn^2+^.

### 4.7. Antioxidant Release from GelMA/ZIF-8 Hydrogels

Antioxidant agents, such as ascorbic acid, can be incorporated into nanocomposite hydrogels. To evaluate the antioxidant activity of the ascorbic acid-loaded nanocomposite hydrogel, a 2,2-Diphenyl-1-picrylhydrazyl (DPPH) assay was performed with some modifications [68]. First, ascorbic acid (10 μg/mL) was added to the lyophilized hydrogels (volume = 150 μL) and allowed to incubate for 5 h. Subsequently, 200 μL of PBS was placed onto the ascorbic acid-loaded nanocomposite hydrogels and left for another 5 h. Different concentrations (10, 15 20, and 25 μg/mL) of the 180 μL DPPH solution were then mixed with the 20 μL of supernatant obtained from the ascorbic acid-loaded nanocomposite hydrogels (n = 5). The resulting mixture was allowed to stand for 30 min at 37 °C before measuring the OD_517_. The percentage scavenging activity was calculated using the following equation:

- Equation (4): % scavenging activity of DPPH

(4) $$\%\;scavenging\;activity\;of\;DPPH=\left[\frac{\left(A0-A1\right)}{A0}\right]*100$$

where A0 represents the absorbance of the control, and A1 represents the absorbance in the presence of the samples.

### 4.8. Model Antibiotic Release from GelMA/ZIF-8 Hydrogel

To analyze the release of model antibiotic, vancomycin (EDM Millipore, USA) from hydrogels, three concentrations of vancomycin (32, 64, and 96 µg/mL) were prepared in PBS and added to each dried 150 µL of GelMA/ZIF-8 hydrogel (n = 3). Each hydrogel was allotted 24 h to allow absorbance of vancomycin. The vancomycin-loaded hydrogels were then placed in dialysis tubing (6–8 kDa molecular weight cut-off) and immersed in 1 mL of PBS at 37.5 °C and placed on a shaker. At desired time intervals, 2 µL of the PBS was collected from the bulk solution surrounding the dialysis tubing for spectroscopic analysis (Spark 20M Plate Reader, Tecan, USA) and analyzed at a wavelength of 281 nm to detect the amount of released vancomycin in the surrounding solution. The collected solution was then returned to the bulk to obtain a cumulative drug release profile. The released vancomycin concentration was determined using a pre-established standard curve. The GelMA/ZIF-8 without vancomycin served as blanks for drug-loaded hydrogels.

- Equation (5): Cumulative drug release (%)

(5) $$Culmuative\;drug\;release\left(\%\right)=\frac{weight\;of\;drug\;released}{weight\;of\;total\;drug}*100$$

### 4.9. Antibacterial Efficacy of GelMA/ZIF-8 Hydrogel

Overnight cultures of *E. coli* DH5α were grown in LB medium (37 °C, 210 RPM). Cultures were pelleted at 13,000 g for 3 min and washed two times with phosphate-buffered saline (PBS), pH 7.0, and resuspended in PBS. The optical density at 600 nm (OD_600_) of this bacterial suspension was determined, and the bacteria were normalized to an OD_600_ equal to 0.1 in PBS. A 1 mL suspension of LB and the experimental groups (GelMA/ZIF-8 hydrogels at different concentration of vancomycin) were inoculated with 10 μL *E. coli* (equal to OD_600_ of 0.01) in 13 mL tubes at 37 °C with 210 RPM shaking for 24 h. The OD_600_ of bacterial cultures were measured for microbial growth. After culturing bacteria exposed to GelMA/ZIF-8 and drug-loaded GelMA/ZIF-8, cultures were diluted to 1 × 10^−6^ in PBS. 100 µL of culture were plated onto LB-agar plates incubated at 37 °C. Following 24 h of incubation, the agar plates were imaged.

### 4.10. Coating Titanium Implants with GelMA/ZIF-8 Hydrogels

#### 4.10.1. Preparation of Gyroid-Shaped Titanium Implants

A cylindrical primitive with the desired outer dimensions (diameter = 12 mm, height = 6 mm) was produced using CAD and rasterized using VTK (Visualization Toolkit, Kitware Inc., Clifton Park, NY, USA) into a binary image volume (voxel size = 0.025 mm isotropic, dimensions = 501 × 501 × 261 voxels). Custom written Python code was used to generate a binary image volume of a sheet-based gyroid lattice of unit-cells (lattice dimensions = 2 × 2 × 1 unit-cells, unit-cell dimension = 6 × 6 × 6 mm) following the procedure outlined in and with a desired porosity of 85% [69]. Wall thickness of the resultant gyroid structure was nominally 0.300 mm and ranged from 0.250–0.350 mm. Image masking of the gyroid lattice binary volume with the cylinder binary volume removed any gyroid lattice structure falling outside the cylinder, thus producing a binary image of a gyroid lattice whose extents had been restricted to fall within confines of the cylinder dimensions. Iso-surfacing the result of the Boolean operation produced a triangulated surface model of the porous cylinder. Surface model triangle count and quality were reduced and improved, respectively, using decimation and remeshing (Geomagic, 3D Systems, Rock Hill, SC, USA). Output of the geometric improvements was an STL file that subsequently was sent for 3D printing in titanium alloy.

Fabrication of the gyroid structure was carried out using laser-powder bed fusion (LPBF) at an additive manufacturing facility (Additive Design in Surgical Solutions, London, ON, Canada) using a commercial 3D metal printer (AM400, Renishaw plc, Wotton-under-Edge, Gloucestershire, UK). Printing parameters were: laser power 200–400 W, scanning speed 10,000–20,000 points/second, particle diameter 15–45 microns, spot size 70 microns. After consolidation, the test components were heat treated (24-h cycle of stress relief, 850 °C for 1 h then passive cool down) to reduce residual stress.

#### 4.10.2. Coating of Gyroids with GelMA/ZIF-8 Hydrogels

Gyroid-shaped implants were dip-coated with GelMA/ZIF-8 pre-gel formulations. Subsequently, the dip-coated implants were exposed to blue-light at 405 nm for 10 min to crosslink the pre-gel. The GelMA/ZIF-8 coated gyroids were then lyophilized and imaged using SEM. Presence of zinc was also confirmed with EDX.

### 4.11. Cyto-Compatibility and Bio-Functionality of GelMA/ZIF-8 Hydrogels

#### 4.11.1. In Vitro Cell Culture

Human adipose-derived stem cells (hASCs; Lot no. 1001002, Gibco, Grand Island, NY, USA) were cultured following the manufacturer’s protocol. Briefly, the cells were incubated at 37 °C in a 5% CO_2_ incubator using MesenPRO RS™ (Gibco, Grand Island, NY, USA) medium supplemented with 2% serum, 1% of p/s, and 200 mM L-glutamine (Thermo Fisher Scientific, Norristown, PA, USA, ca 25030081). The medium was replaced every 3 days, and the cells were harvested using TrypLE™ Express without phenol red (Gibco, Grand Island, NY, USA) and resuspended in fresh culture medium. Osteogenic medium was prepared in MesenPRO RS™ supplemented with 1% p/s and 50 µM ascorbic acid (Sigma, Ronkonkoma, NY, USA), 100 nM Dexamethasone, and 10 mM ß-glycerophosphate (Sigma, Ronkonkoma, NY, USA).

#### 4.11.2. Cell Proliferation and Cyto-Compatibility

To determine cell proliferation, 3-(4,5-dimethylthiazol-2-yl)-5-(3-carboxymethoxyphenyl)-2-(4-sulfophenyl)-2H-tetrazolium (MTS; Promega, USA) assay was performed following the manufacturer’s instructions. Briefly, the hydrogels were placed in a 96-well plate and hASCs were seeded onto the hydrogels at a seeding density of 1 × 10^4^ cells per well. The cells were incubated for 24 h after which the culture media was replaced with 20 µL of MTS reagent containing 100 µL of fresh culture media. The plate was then incubated at 37 °C for 2 h and the absorbance was measured at 490 nm using a 96-well plate reader (Asys UVM 340 Scanning Microplate Reader, Biochrom Ltd., Cambridge, UK). Lastly, the morphology of the cells was visualized by differential interference contrast (DIC) microscopy (IX81, Olympus, Westborough, MA, USA).

#### 4.11.3. Determining Cell Viability by Calcein Acetoxymethyl (Calcein-AM) Staining

To determine cell viability in the presence of GelMA/ZIF-8 hydrogels, calcein-AM (Thermo Fisher, Ronkonkoma, NY, USA) was performed following the manufacturer’s instructions. 20 µL of GelMA hydrogel with various concentrations of ZIF-8 nanoparticles were placed into a 96-well plate. The pre-gel was then crosslinked using visible light (405 nm) at 10-min exposure. The crosslinked hydrogels were then washed three times with fresh PBS for 18 h to remove unreacted components and tartrazine. hASCs were seeded onto the crosslinked hydrogel at a density of 1 × 10^4^ cells per well. On day 1, 2 µM calcein AM was added to hASCs and incubated for 20 min at 37˚C. The live cells were imaged using fluorescence microscopy (IX81, Olympus, Westborough, MA, USA).

#### 4.11.4. Determining Osteogenic Differentiation of Stem Cells Using Alizarin Red S (ARS) Staining

Cells were fixed with 4% formalin for 10 min and then washed three times with PBS. Alizarin red S solution (ARS, ScienCell, ARS Staining Quantification Assay, Catalog No. 8678, USA) was added for 30 min at room temperature and then the fixed cells were washed with distilled water to remove excess of ARS solution. The ARS staining was imaged using Nikon Eclipse Microscope (Nikon, Tokyo, Japan) and processed using the NIS elements software from Nikon. To quantify the ARS, 10% acetic acid was added to the cells. After 10 min of incubation at 85 °C, the cells with the acetic acid were centrifuged for 15 min at 20,000× *g*. The supernatant was collected to other tubes and neutralized with 10% ammonium hydroxide. 150 µL of each sample was added to a 96-well plate and the OD_405_ was read using Spark^®^ multimode microplate reader from Tecan, San Jose, CA, USA.

#### 4.11.5. Quantifying the Ability of GelMA/ZIF-8 Hydrogels to Promote Osteogenic Differentiation of Stem Cells Using Real-Time Quantitative Polymerase Chain Reaction (RT-qPCR) Analysis

Stem cells were cultured with experimental groups (control, 3D printed GelMA and GelMA/ZIF-8 hydrogls) in 24-well plates in osteogenic medium for 21 days. Total RNA was isolated using RNeasy Micro Kit (Qiagen, Hilden, Germany) and 150 µg RNA was reversed transcribed to cDNA using High-Capacity cDNA Reverse Transcription Kit (Thermo Fisher Scientific, Norristown, PA, USA) according to the manufacturer’s instructions. Gene expression was quantified by using TB Green Advantage qPCR premix (Takara, San Jose, CA, USA). The expression of genes, including osteopontin (OPN) and runt-related transcription factor-2 (RUNX2) which are known as the expression of a regulator of osteoblast differentiation. Real-time PCR analysis was repeated three trials for each group. The gene expression level of each target gene was calculated by the ΔΔC_T_ method and was normalized to that of the reference gene glyceraldehyde 3-phosphate dehydrogenase (GAPDH). The primers sequences for the selected genes are shown in Table 3.

### 4.12. Evaluating Cell Viability in 3D Bio-printing through Live/Dead Cell Staining

To evaluate the cytotoxicity of nanocomposite hydrogels, a Live/Dead cell imaging assay (Invitrogen by Thermo Fisher Scientific, Cat. No. R37601, Norristown, PA, USA) was carried out. Briefly, hASCs were harvested using trypsin, and then they were gently mixed with the pre-GelMA solution. Cells were seeded at a density of 1 × 10^4^ cells per well in a 96-well plate. After completing the crosslinking, we wash the hydrogels for 10 min with culture media at 37 °C and 5% CO_2_. This step is essential for removing any unreacted component and improving cell viability. Following this, hydrogels are cultured in fresh culture media. The cells were then stained with the Live/Dead cell imaging kit for 1 day, 3 days, and 7 days.

### 4.13. Statistical Analysis

One-way ANOVA was used to determine statistical significance for studies with more than two groups tested, such as cell proliferation study using MTS assay and RT-qPCR analysis, and Tukey procedure for post hoc comparison. *p* < 0.05 was considered statistically significant. A *t*-test was carried out when comparing the mean of two sets of data, for example, the quantification of the compressive module of GelMA and GelMA/ZIF-8 hydrogels.

## Figures and Tables

**Table 1:** Amount of 10 mg/mL ZIF-8 solution required to be dried to achieve desired ZIF-8 concentration in a final 300 µL GelMA hydrogel formulation.

Desired ZIF-8 Conc. in GelMA Hydrogel Formulation (mg/mL)	Amount of 10 mg/mL ZIF-8 Solution Required (µL)
0.5	15
1	30
2	60
3	90

**Table 2:** Preparation of polymeric hydrogel for 3D bio-printing.

Components	Stock Conc. (% *w*/*v*)	Component Conc. in Hydrogel (% *v*/*v*)		
GelMA	20	5	10	15
LAP	8	1	1	1
Tartrazine	1	0.1	0.1	0.1

**Table 3:** Primer sequences used for RT-qPCR.

Gene	Species	Forward Primer Sequence (5′-3′)	Reverse Primer Sequence (5′-3′)	Ref.
OPN	Human	GTGCAGAGGAAACCGAAGAG	TGTTTGCAGTGGTGGTTCTG	[70]
RUNX2	Human	CCCGTGGCCTTCAAGGT	CGTTACCCGCCATGACAGTA	[70]
GAPDH	Human	AACAGCACCCACTCCTC	CATACCAGGAAATGAGCTTGACAA	[71]

## Data Availability

The data presented in this study are openly available in article.

## Supplementary Materials

The following supporting information can be downloaded at: https://www.mdpi.com/article/10.3390/gels9120923/s1, Figure S1: Physicochemical characteristic of ZIF-8 nanoparticles. (A) The UV-Vis spectra analysis of ZIF-8 nanoparticles in water revealed that the maximum absorption consistently occurred at 215 nm in different concentrations. (B) Visual representation the zinc ions release from ZIF-8 nanoparticles. ZIF-8 undergoes degradation, resulting in the release of Zn ions and 2-Melm, in response to surrounding anions and acidic conditions. (C) Zn^2+^ release standard curve measured by a spectrophotometric method (n = 3); Figure S2: Optimizing biomaterial formulation via 3D printing. (A) Printability of GelMA and GelMA/ZIF-8 nanocomposite hydrogels at varying compositions. (B) Corresponding images of printable hydrogels. (C) Images of unprintable hydrogels. Unprintable hydrogels were classified by their inability to retain their shape and meet the dimensions of the desired structure (4 mm diameter, 2 mm height). (D) Corresponding images of printable hydrogels. Corresponding height of printable hydrogels (n = 3). (E) Corresponding diameter of printable hydrogels (n = 3); Figure S3: Optimizing the printing parameters (power and intensity) of the LumenX+ DLP bioprinter. (A) Corresponding images of hydrogels printed using various power (%) and time exposure (seconds). (B) Corresponding height of hydrogels (n = 3). (C) Corresponding diameter of hydrogels (n = 3); Figure S4: The incorporation of antioxidant agents into nanocomposite hydrogels significantly enhanced their antioxidant activity. A nanocomposite hydrogel was loaded with 10 μg/mL of ascorbic acid as an antioxidant agent (n = 3). The antioxidant-loaded nanocomposite hydrogel demonstrated diverse scavenging activities in relation to different concentrations of DPPH. The results indicated that as the concentrations of the DPPH agents increased, the efficacy of %scavenging further improved, providing confirmation of antioxidant activity in the nanocomposite hydrogel. * *p*-value < 0.05, ** *p*-value < 0.01, *** *p*-value < 0.001, and **** *p*-value < 0.0001; Figure S5: Live/dead cell viability assay of hADS cells cultured with the crosslinked GelMA/ZIF-8. The cells were cultured for 1 day, 3 day, and 7 days and then stained with the Live/Dead cell imaging staining kit. The live and dead cells exhibited green and red fluorescence, respectively. The scale bar represents 100 μm.
