# Supplementary material for: Metal Organic Framework-Incorporated Three-Dimensional (3D) Bio-Printable Hydrogels to Facilitate Bone Repair: Preparation and In Vitro Bioactivity Analysis
Source: Gels. 2023 Nov 23;9(12):923. doi: 10.3390/gels9120923 (PMC10742699; doi:10.3390/gels9120923)
Supplement: Supplementary file 1 [file gels-09-00923-s001.zip › gels-2687498-supplementary.pdf]

Supporting Information

Metal Organic Framework-Incorporated Three-Dimensional (3D) Bio-printable Hydrogels to Facilitate Bone Repair: Preparation and In Vitro Bioactivity Analysis

Cho-E Choi [1], Aishik Chakraborty [1,2], Hailey Adzija [3], Yasmeen Shamiya [3], Khaled Hijazi [2,4], Ali Coyle [4], Amin Rizkalla [1,2,4,5,6], David Holdsworth [5], and Arghya Paul [1,2,3,4]*

[1] Department of Chemical and Biochemical Engineering, The University of Western Ontario, London, ON N6A 5B9, Canada

[2] Collaborative Specialization in Musculoskeletal Health Research and Bone and Joint Institute, The University of Western Ontario, London, ON N6A 5B9, Canada;

[3] Department of Chemistry, The University of Western Ontario, London, ON N6A 5B9, Canada

[4] School of Biomedical Engineering, The University of Western Ontario, London, ON N6A 5B9, Canada

[5] Department of Medical Biophysics, The University of Western Ontario, London, ON N6A 5B9, Canada

[6] Dentistry, The University of Western Ontario, London Ontario, London ON N5A 5B9, Canada

* Correspondence: arghya.paul@uwo.ca; Tel.: 519-661-2111

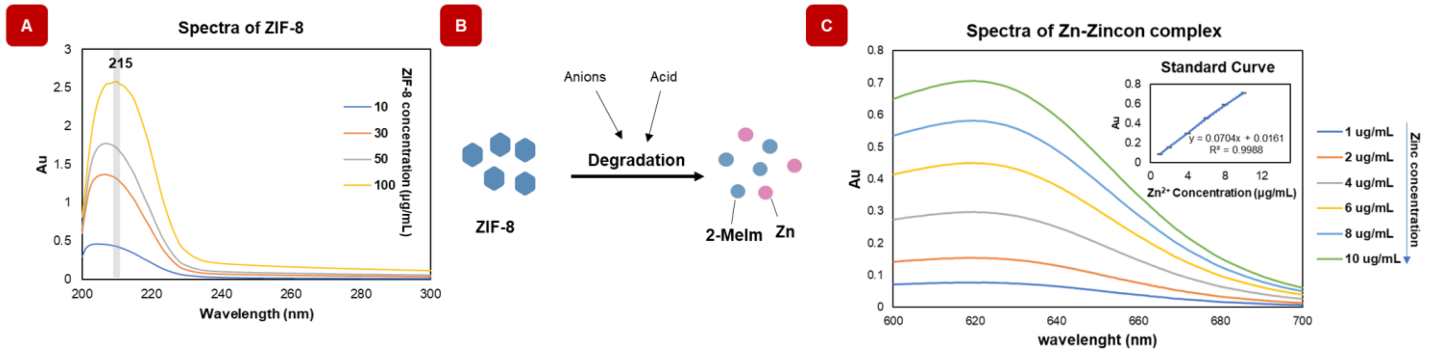

Figure S1. Physicochemical characteristic of ZIF-8 nanoparticles. A) The UV-Vis spectra analysis of ZIF-8 nanoparticles in water revealed that the maximum absorption consistently occurred at 215 nm in different concentrations. B) Visual representation the zinc ions release from ZIF-8 nanoparticles. ZIF-8 undergoes degradation, resulting in the release of Zn ions and 2-Melm, in response to surrounding anions and acidic conditions. C) Zn^{2+} release standard curve measured by a spectrophotometric method. (n = 3)

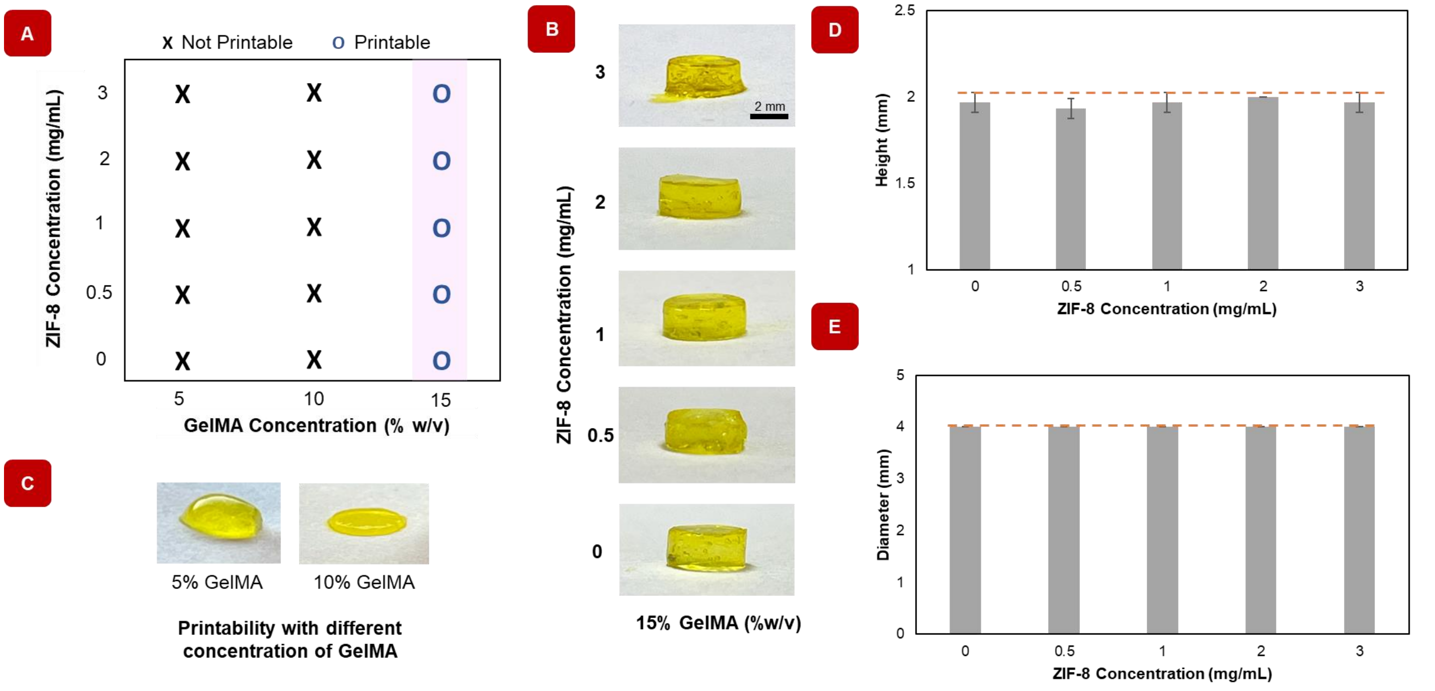

Figure S2. Optimizing biomaterial formulation via 3D printing. A) Printability of GelMA and GelMA/ZIF-8 nanocomposite hydrogels at varying compositions. B) Corresponding images of printable hydrogels. C) Images of unprintable hydrogels. Unprintable hydrogels were classified by their inability to retain their shape and meet the dimensions of the desired structure (4 mm diameter, 2 mm height). D) Corresponding images of printable hydrogels. D) Corresponding height of printable hydrogels (n = 3). E) Corresponding diameter of printable hydrogels (n = 3).

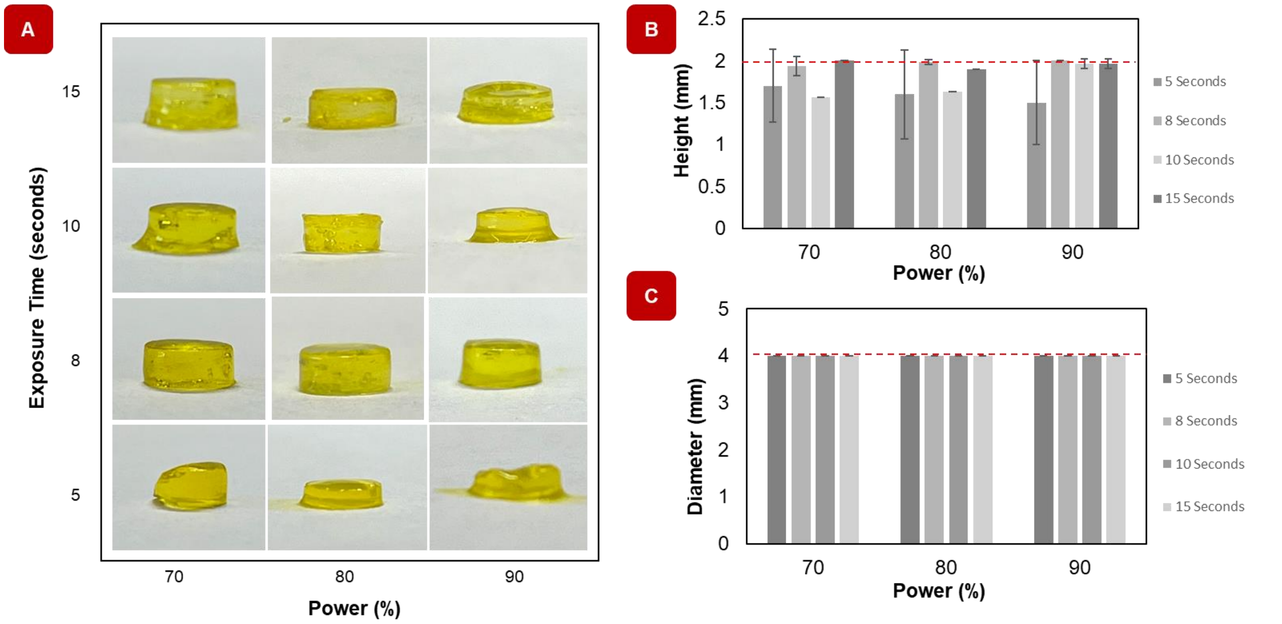

Figure S3. Optimizing the printing parameters (power and intensity) of the LumenX+ DLP bioprinter. A) Corresponding images of hydrogels printed using various power (%) and time exposure (seconds) B) Corresponding height of hydrogels (n = 3). C) Corresponding diameter of hydrogels (n = 3).

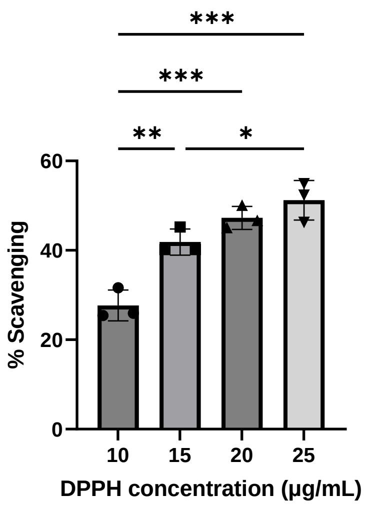

Figure S4. The incorporation of antioxidant agents into nanocomposite hydrogels significantly enhanced their antioxidant activity. A nanocomposite hydrogel was loaded with 10 μg/mL of ascorbic acid as an antioxidant agent (n = 3). The antioxidant-loaded nanocomposite hydrogel demonstrated diverse scavenging activities in relation to different concentrations of DPPH. The results indicated that as the concentrations of the DPPH agents increased, the efficacy of %scavenging further improved, providing confirmation of antioxidant activity in the nanocomposite hydrogel. *p-value < 0.05, **p-value < 0.01, ***p-value < 0.001, and ****p-value < 0.0001.

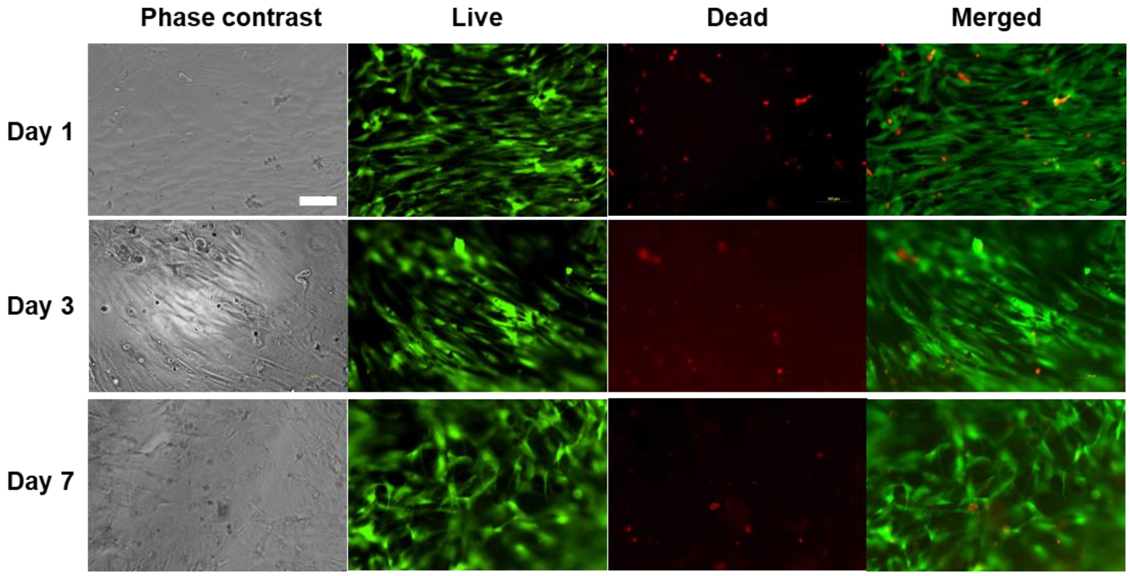

Figure S5. Live/dead cell viability assay of hADS cells cultured with the crosslinked GelMA/ZIF-8. The cells were cultured for 1 day, 3 day, and 7 days and then stained with the Live/Dead cell imaging staining kit. The live and dead cells exhibited green and red fluorescence, respectively. The scale bar represents 100 µm.
